# Expression of the HMGI(Y) gene products in human neuroblastic tumours correlates with differentiation status

**DOI:** 10.1054/bjoc.2000.1494

**Published:** 2000-11-22

**Authors:** G Giannini, C J Kim, L Di Marcotullio, G Manfioletti, B Cardinali, F Cerignoli, E Ristori, M Zani, L Frati, I Screpanti, A Gulino

**Affiliations:** Department of Experimental Medicine and Pathology, University La Sapienza, Rome, 00161; Department of Pathology, Seoul National University College of Medicine, Korea; Department of Biochemistry, Biophysics and Chemistry of the Macromolecules, University of Trieste; Istituto di Biologia Cellulare, CNR, Rome; Department of Experimental Medicine, University of L'Aquila, L'Aquila, 67100; IMN, Neuromed Institute, Pozzili, Italy

**Keywords:** HMGI, neuroblastic tumours, retinoic acid

## Abstract

HMGI and HMGY are splicing variants of the HMGI(Y) gene and together with HMGI-C, belong to a family of DNA binding proteins involved in maintaining active chromatin conformation and in the regulation of gene transcription. The expression of the HMGI(Y) gene is maximal during embryonic development, declines in adult differentiated tissues and is reactivated in most transformed cells in vitro and in many human cancers in vivo. The HMGI(Y) genomic locus is frequently rearranged in mesenchymal tumours, suggesting a biological role for HMGI(Y) gene products in tumour biology. HMGIs are both target and modulators of retinoic acid activity. In fact, HMGI(Y) gene expression is differentially regulated by retinoic acid in retinoid-sensitive and -resistant neuroblastoma cells, while HMGI-C participates in conferring retinoic acid resistance in some neuroblastoma cells. In this paper we show that HMGI and HMGY isoforms are equally regulated by retinoic acid in neuroblastoma cell lines at both RNA and protein levels. More importantly our immunohistochemical analysis shows that, although HMGI(Y) is expressed in all neuroblastic tumours, consistently higher levels are observed in less differentiated neuroblastomas compared to more differentiated ganglioneuromas, indicating that HMGI(Y) expression should be evaluated as a potential diagnostic and prognostic marker in neuroblastic tumours. © 2000 Cancer Research Campaign http://www.bjcancer.com


*British Journal of Cancer* (2000) **83**(11), 1503–1509
© 2000 Cancer Research Campaign
doi: 10.1054/ bjoc.2000.1494, available online at http://www.idealibrary.com on IDEAL®

### Expression of the HMGI(Y) gene products in human neuroblastic tumours correlates with differentiation status

**G Giannini[1], CJ Kim[2], L Di Marcotullio[1], G Manfioletti[3], B Cardinali[4], F Cerignoli[5], E Ristori[1], M Zani[1], L Frati[1,6], I Screpanti[1] and A Gulino[1,6]**

[1]Department of Experimental Medicine and Pathology, University La Sapienza, 00161 Rome; [2]Department of Pathology, Seoul National University College of Medicine, Korea; [3]Department of Biochemistry, Biophysics and Chemistry of the Macromolecules, University of Trieste; [4]Istituto di Biologia Cellulare, CNR, Rome; [5]Department of Experimental Medicine, University of L'Aquila, 67100 L'Aquila; [6]IMN, Neuromed Institute, Pozzili, Italy

Summary HMGI and HMGY are splicing variants of the HMGI(Y) gene and together with HMGI-C, belong to a family of DNA binding proteins involved in maintaining active chromatin conformation and in the regulation of gene transcription. The expression of the HMGI(Y) gene is maximal during embryonic development, declines in adult differentiated tissues and is reactivated in most transformed cells in vitro and in many human cancers in vivo. The HMGI(Y) genomic locus is frequently rearranged in mesenchymal tumours, suggesting a biological role for HMGI(Y) gene products in tumour biology. HMGIs are both target and modulators of retinoic acid activity. In fact, HMGI(Y) gene expression is differentially regulated by retinoic acid in retinoid-sensitive and -resistant neuroblastoma cells, while HMGI-C participates in conferring retinoic acid resistance in some neuroblastoma cells. In this paper we show that HMGI and HMGY isoforms are equally regulated by retinoic acid in neuroblastoma cell lines at both RNA and protein levels. More importantly our immunohistochemical analysis shows that, although HMGI(Y) is expressed in all neuroblastic tumours, consistently higher levels are observed in less differentiated neuroblastomas compared to more differentiated ganglioneuromas, indicating that HMGI(Y) expression should be evaluated as a potential diagnostic and prognostic marker in neuroblastic tumours. © 2000 Cancer Research Campaign http://www.bjcancer.com

**Keywords:** HMGI; neuroblastic tumours; retinoic acid

Neuroblastoma (NB), ganglioneuroblastoma (GNB) and ganglioneuroma (GN) are paediatric tumours arising from a common neural crest derived precursor cell which fails to undergo complete differentiation into neurons of the sympathetic ganglia or chromaffin cells of the adrenal medulla. Several parameters including age, proliferation rate, ploidy, *MYCN* amplification, chromosome 1p deletion and Trk receptors' expression definitely help for a more precise clinicopathological characterization of these neoplasias. However most of the different classifications proposed for neuroblastic tumours take into great account the histologic appearance (Castleberry, 1997; Katzenstein et al, 1998; Shimada et al, 1999). In fact, a high degree of ganglionic differentiation observed in GN is usually associated with a non-aggressive phenotype. In contrast, NB with undifferentiated histology reflects a more aggressive behaviour in most cases. Interestingly, at variance with most neoplasia of the adult, neuroblastoma tumours retain in many cases the ability to differentiate (i.e., towards a neuronal phenotype) in vivo (Bolande, 1985). Differentiation of NB cell cultures towards a neuronal phenotype can also be obtained in vitro by means of several factors (Albemayor and Sidell, 1989; Thiele, 1991a). Probably because of its potential therapeutic value, retinoic acid (RA) is one of the most frequently used inducers of NB cell differentiation. RA operates through three different RXR/RAR heterodimers to transduce its signals in NB cells (Giannini et al, 1997).

HMGI and HMGY isoforms originate from an alternative splicing of the same gene located on chromosome 6p21. The two proteins only differ by the exclusive presence of 11 amino acids in the HMGI molecule, that are supposed to act as a spacer between the first and the second of the three 'AT-hook' DNA binding domains (Johnson et al, 1989; Friedmann et al, 1993). We will refer to this gene and to its gene products as HMGI(Y), unless we want to discriminate between HMGI and HMGY isoforms. A third HMGI family member, HMGI-C, is encoded by a different gene (Manfioletti et al, 1991) which maps to chromosome 12q13–15 (Schoenmakers et al, 1995; Ishwad et al, 1997). The three proteins are considered architectural transcription factors and are involved in chromatin conformation functions and in regulating transcription through several gene promoters (reviewed in Bustin and Reeves, 1996). Expression of the *HMGI* gene family is prominent during early stages of mammalian development, where they are likely to play an important role in the regulation of cell proliferation and differentiation (Zhou et al, 1995; Chiappetta et al, 1996; Hirning-Folz et al, 1998). This is clearly suggested by the fact that deletions of the *HMGI-C* gene are responsible for the *pigmy* phenotype in mouse. This is characterized by a reduced body size (40% than normal) and a cell autonomous growth defect (Zhou et al, 1995). Conversely, transgenic mice expressing a truncated version of the HMGI-C protein display a *giant* phenotype (Battista et al, 1999) and a high incidence of lipoma (Arlotta et al, 2000).

*Received 4 April 2000*
*Revised 19 July 2000*
*Accepted 14 August 2000*

*Correspondence to:* G Giannini

Although in mammals most adult differentiated tissues express no *HMGI-C* and little or no *HMGI(Y)* (Zhou et al, 1995; Chiappetta et al, 1996; Hirning-Folz et al, 1998), wild-type or anomalous HMGI proteins are heavily expressed in several benign and malignant neoplasia. *HMGI-C* gene is frequently rearranged in most benign mesenchymal tumours, and rearranged or amplified in sarcomas and leukaemias (Schoenmakers et al, 1995; Kazmierczak et al, 1996; Berner et al, 1997; Rommel et al, 1997; Kottickal et al, 1998; Pedeutour et al, 2000). High levels of *HMGI(Y)* expression have been identified in a number of transformed cell lines (Giancotti et al, 1987). In vitro studies have shown that impairment of the expression of HMGI proteins prevents oncoviral transformation of rat thyroid cells (Giancotti et al, 1987; Berlingieri et al, 1995), thus establishing a strong link between high level of *HMGI(Y)* expression and transformed phenotype. A number of studies have shown that *HMGI(Y)* expression is increased in human epithelial tumours including thyroid, colorectal, uterine cervix and prostate cancer (Chiappetta et al, 1995; Fedele et al, 1996; Tamimi et al, 1996; Bandiera et al, 1998). Extensive analysis of thyroid and colorectal tumours brought to the conclusion that quantitation of the level of *HMGI(Y)* expression in tissue sections and/or RNA samples might provide a potential diagnostic indicator (Chiappetta et al, 1998; Abe et al, 1999). In contrast, scant attention has been paid to the biological role of HMGI proteins in the nervous system and to the potential significance of their expression for neural cell tumorigenesis.

In a previous study, we have addressed the question whether expression of *HMGI* genes is altered in neuroblastic tumours. We have shown that HMGI-C mRNA is expressed in a subset of NB tumour specimens and only in NB cell lines resistant to the antiproliferative and differentiative effects of RA (Giannini et al, 1999). HMGI-C has also been shown to impair RA-responsiveness, since its exogenous expression counteracts RA-mediated growth inhibition in SY5Y NB cells (Giannini et al, 1999). In contrast, *HMGI(Y)* expression was detected in all NB tumours and cell lines, although it is differentially regulated by RA in RA-sensitive and -resistant cells. Thus, expression or regulation of *HMGI* genes might be related to NB cell differentiation and growth. In this paper we have extended these results by showing that HMGI and HMGY isoforms are both expressed at the mRNA and protein level in all NB cell lines and that both are further upregulated by RA in RA-resistant NB cells. More interestingly, we also show higher immunohistochemical expression of HMGI(Y) protein in undifferentiated neuroblasts populating NB tumours compared to more differentiated GNB and GN. Our results suggest that *HMGI(Y)* expression should be regarded as a potential diagnostic indicator of lack of differentiation of neuroblastic tumours.

### MATERIALS AND METHODS

### Cell culture conditions

The human neuroblastoma cell lines SK-N-AS, SH-EP, KCNR, SK-N-BE and SY5Y were cultured in RPMI 1640 medium (or E-MEM for SK-N-SH) supplemented with 10% fetal bovine serum (FBS), 2 mM glutamine, 50 units ml$^{-1}$ penicillin, and 50 µg ml$^{-1}$ streptomycin at 37°C with 5% $CO_2$. For stimulation experiments cells were seeded at low density. *All-trans* retinoic acid (Sigma Chemicals Co, St Louis, MO, 1 µM) was added 24 hours after seeding. On the basis of a time course analysis previously reported (Giannini et al, 1999), the clearest results were observed after 24 hours of treatment for SK-N-AS, SH-EP and SK-N-SH. 48 hours of treatment were required to observe HMGI(Y) repression in KCNR and SY5Y cells, while much longer treatment (10–14 days) was required for SK-N-BE cells.

### RNA preparation and RT-PCR

Following treatment, cells were scraped from the dish, washed twice with ice-cold PBS and processed for RNA extraction with the RNAeasy kit (Quiagen Inc, Chatsworth, CA) according to the manufacturer's instructions. The RT reaction was performed on total RNA (1 µg) using the Moloney murine leukaemia virus reverse transcriptase kit according to the manufacturer's instructions (Life Technologies, Inc) in a final volume of 20 µl for 45 min at 42°C. An aliquot (2 µl) of the RT reaction was subjected to a semiquantitative PCR reaction (1 min at 94°C, 1 min at 58°C, 2 min at 72°C, for 20, 25 or 30 cycles) for HMGI(Y) amplification with the specific primers HMGI(Y)#1, 5′-CTGCTCCTCCTCC-GAGGAC-3′ and HMGI(Y)#2 5′-AAGATGAGTGAGTC-GAGCTC-3′, PCR products were electrophoresed on a 2.5% Nusieve agarose gel and blotted on hybridization membranes (Gene Screen Plus, NEN Life Science Product, Boston, MA). A second PCR fragment amplified by using primers HMGI(Y)#PR, 5′-CCTCCTTCACTGTTCCCTCT-3′ and HMGI(Y)#3, 5′-TAC-GGGGACTAGGGAAGTTG-3′, was cloned, $^{32}$P-labelled and subsequently used as internal probe for the hybridization of NB tumour samples. 1/40 of the same RT reaction was subjected to a semiquantitative PCR (1 min at 94°C, 1 min at 58°C, 2 min at 72°C, for 20, 25 or 30 cycles) to quantitatively amplify the β-actin cDNA as a control using the specific primers: β-act#3, 5′-CTACAATGAGCTGCGTGTGG-3′; β-act#4, 5′-CGGTGAG-GATCTTCATGAGG-3′). Amplicons were electrophoresed, blotted on nylon membranes and hybridized to a β-actin specific probe.

### Protein extraction and Western blot analysis

Total HMG proteins were selectively extracted by 5% (v/v) perchloric acid from cells cultured as above and analysed by Western blot as previously reported (Bandiera et al, 1998). Equal amounts of proteins from the different control or RA-treated cell lines were run on a 15% SDS-polyacrylamide gel in Tris/ricine/SDS buffer. Coomassie staining of the higher parts of the gels allowed the detection of the histone H1, that can be assumed to be constantly expressed in different cell lines (Giancotti et al, 1993) and can be used to confirm the equal loading of the wells (Bandiera et al, 1998). The lower part of the gels was transferred by semi-dry blotting onto 0.1 µM-pore PVDF Millipore membranes. They were subsequently blocked in 5% non-fat dry milk and incubated with rabbit polyclonal antibody developed against a peptide derived from HMGI protein (Chiappetta et al, 1995; Bandiera et al, 1998). For the detection of specifically recognized proteins, we took advantage of the SuperSignal Substrate supplied by Pierce (Rockford, IL).

### Immunohistochemistry of HMGI(Y)

A total of 11 cases of neuroblastic tumours were selected from the file of the Department of Pathology, Seoul National University Children's Hospital, and 5 µm-thick sections were obtained from

*© 2000 Cancer Research Campaign*

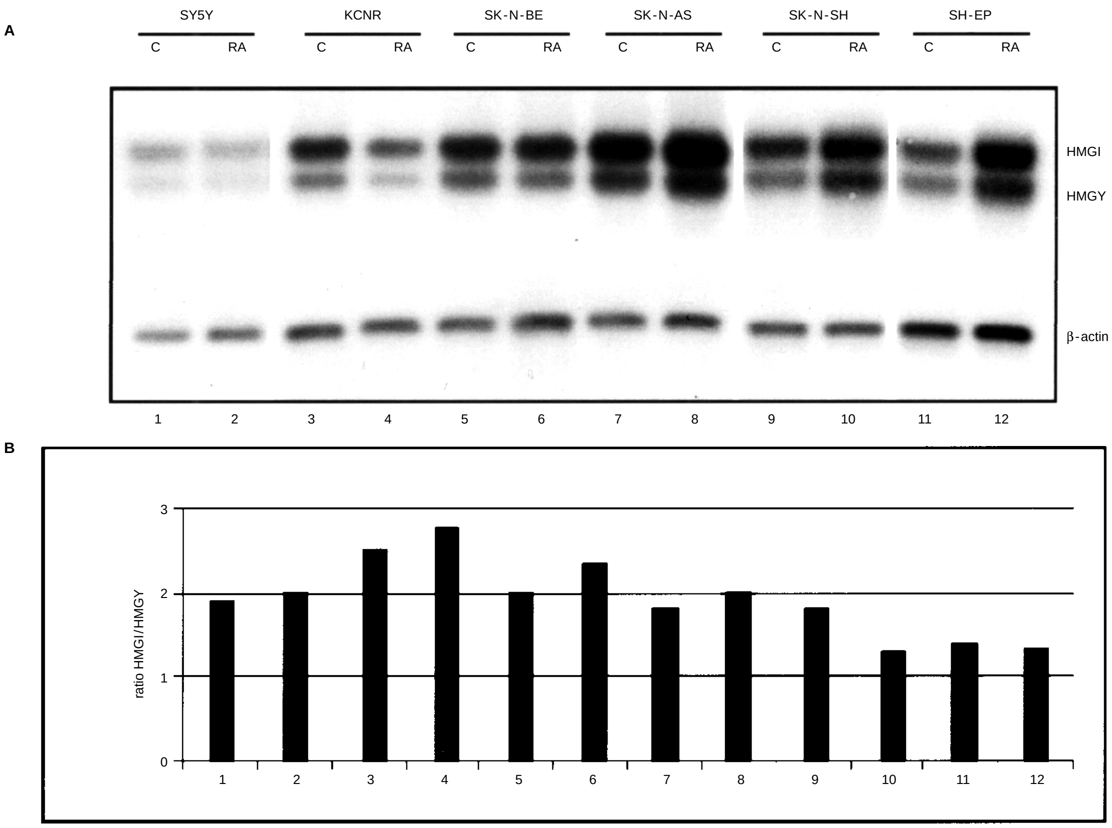

**Figure 1** RT-PCR analysis of HMGI and HMGY expression in NB cell lines. RT-PCR analysis was performed on total RNA extracted from control or RA-treated NB cells by amplification of the entire coding sequence and electrophoretic run on a 2.5% agarose gel. The amplification products were then blotted and hybridized to a specific HMGI(Y) probe as reported in material and methods. (**A**) Exemplification of one of three different RT-PCR amplifications performed on the same RNAs in which HMGI and HMGY amplicons can be clearly distinguished after hybridization. (**B**) After the densitometric scanning of the X-ray films, the ratio between HMGI and HMGY intensity values was calculated for each lane and has been reported graphically. HMGI intensity is on average 2 times HMGY and the ratio is only poorly affected by RA in most NB cell lines.

representative paraffin blocks. Sections were deparaffinized and hydrated with xylene and graded alcohol respectively. Endogenous peroxidase was quenched with 3.0% hydrogen peroxide in ethanol for 10 minutes. After PBS rinses, the sections were incubated overnight at 4°C with 1:100 diluted rabbit anti-HMGI(Y) (Chiappetta et al, 1995; Bandiera et al, 1998). Detection was performed using a large volume DAKO LSAB kit (DAKO A/S, Glostrup, DK). Linking agent (the biotinylated anti-rabbit and anti-mouse immunoglobulins contained in the kit and diluted in PBS) and streptavidin conjugated to horseradish peroxidase in Tris-HCl buffer were sequentially applied for 30 minutes. PBS rinsing was performed between each step. 3,3′-diaminobenzidine was used as chromogen, and counterstaining was done with Meyer's haematoxylin. Immunoreactivity was graded according to the fraction of positive undifferentiated or neuronal cells; +: >10–30%, ++: 30–60%, +++: ≥60%.

### RESULTS

### Expression of HMGI and HMGY isoforms in NB cell lines

We have recently reported the expression of the *HMGI(Y)* and *HMGI-C* gene in human neuroblastoma cell lines and tumours using Northern blot and RT-PCR. In addition, we have also reported that *HMGI(Y)* expression can be modulated by retinoic acid (Giannini et al, 1999). However, none of the assays we previously used could reveal which of the two isoforms of the gene, namely HMGI or HMGY, was expressed in NB and modulated by RA. Therefore we performed RT-PCR on total RNA extracted from RA-treated or control NB cells, using two primers which could amplify the entire coding sequence of the *HMGI(Y)* gene under exponential amplification conditions. The PCR products were electrophoretically run, blotted and hybridized to a specific probe. Even though the modifications of *HMGI(Y)* expression in response to RA were better appreciated by a more quantitative methodology as Northern blot (Giannini et al, 1999), under the present experimental conditions we could easily discriminate between the two isoforms (Figure 1A). One of three identical experiments we performed is illustrated in Figure 1. The data confirmed and extended our previous results, since we found that both HMGI and HMGY are clearly expressed in all the cell lines analysed (Figure 1). We also confirmed that their expression could be positively modulated by RA in SK-N-AS, SK-N-SH and SH-EP cells which fail to undergo growth arrest and differentiation following RA treatment (Giannini et al, 1999). Conversely, HMGI and HMGY isoforms are slightly repressed by RA in SY5Y, KCNR and SK-N-BE cells (Figure 1A). Densitometric analysis of the blots indicated that HMGI transcript expression is on average 2 fold higher than HMGY (Figure 1B). Although RA treatment did not induce high variations in the ratio between the two isoforms, some minor differences could be noted. It appeared that the ratio

© 2000 Cancer Research Campaign

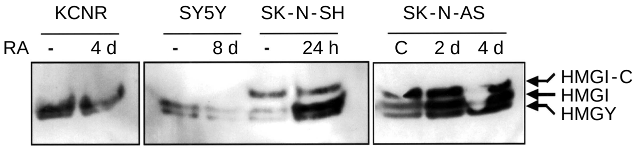

**Figure 2** Western blot analysis of HMGI and HMGY expression in NB cell lines. Appropriate electrophoretic and blotting conditions were used to resolve HMGI and HMGY bands. Equal loading of the lanes was confirmed by the equal intensity of the histone H1 bands observed in the Coomassie-stained upper portion of the gels (see Materials and methods). The membranes were probed with the specific anti-HMGI(Y) antibody and the two isoforms can be clearly distinguished. HMGI and HMGY expression was analysed at different time points after RA treatment. Only informative time points are reported; 24 hours of RA treatment are enough to detect a strong increase of both HMGI and HMGY in SK-N-SH cells. A sustained increase in both proteins is observed in 2 and 4 days RA-treated SK-N-AS cells. Only a slight decrease in HMGI and HMGY proteins could be detected in KCNR and SY5Y cells even after longer exposure to RA. The same membranes were also used to assess HMGI-C expression, which, as expected is not expressed in SY5Y and KCNR cells and is constitutively expressed in SK-N-AS and SK-N-SH cells.

between HMGI versus HMGY expression is slightly increased by RA in KCNR, SK-N-BE, SK-N-AS cell lines, did not vary in SY5Y and SH-EP, and was slightly reduced in SK-N-SH (Figure 1B).

The regulation of HMGI and HMGY expression by RA was also studied at the protein expression level by Western blot. The specific polyclonal antibodies (Chiappetta et al, 1995; Bandiera et al, 1998) detected both HMGI and HMGY expression in all the cell lines (Figure 2). In SK-N-SH, we detected a clear increase of both HMGI and HMGY isoforms after 1 day of RA treatment and SK-N-AS showed sustained increase in both isoforms between 2 and 4 days of RA treatment (Figure 2). In contrast we only observed very minor variations of HMGI and HMGY protein expression in KCNR and SY5Y cells (Figure 2). The same membranes were subsequently probed with anti-HMGI-C specific antibodies. As much as its RNA counterpart (Giannini et al, 1999), HMGI-C protein was absent in KCNR and SY5Y and constitutively expressed in SK-N-AS and SK-N-SH, where it was not affected by RA (Figure 2).

### Expression of HMGI(Y) in neuroblastic tumours

Besides its expression in NB cell lines, we have recently observed the expression of HMGI(Y) transcripts in 23 out of 23 (100%) neuroblastoma cases, through a sensitive RT-PCR approach (Giannini et al, 1999). More recently we have extended this analysis to a total of 65 samples, confirming *HMGI(Y)* expression in all the RNA samples extracted from NB tumour specimens (not shown). However this method did not allow us to determine which of the different cell types commonly present within the tumour mass is responsible for *HMGI(Y)* expression, neither was it compatible with an accurate quantitative analysis of its expression. To gain such information and to provide more stringent evidence on *HMGI(Y)* expression, we have used specific antibodies to perform immunohistochemistry on a number of neuroblastic tumours.

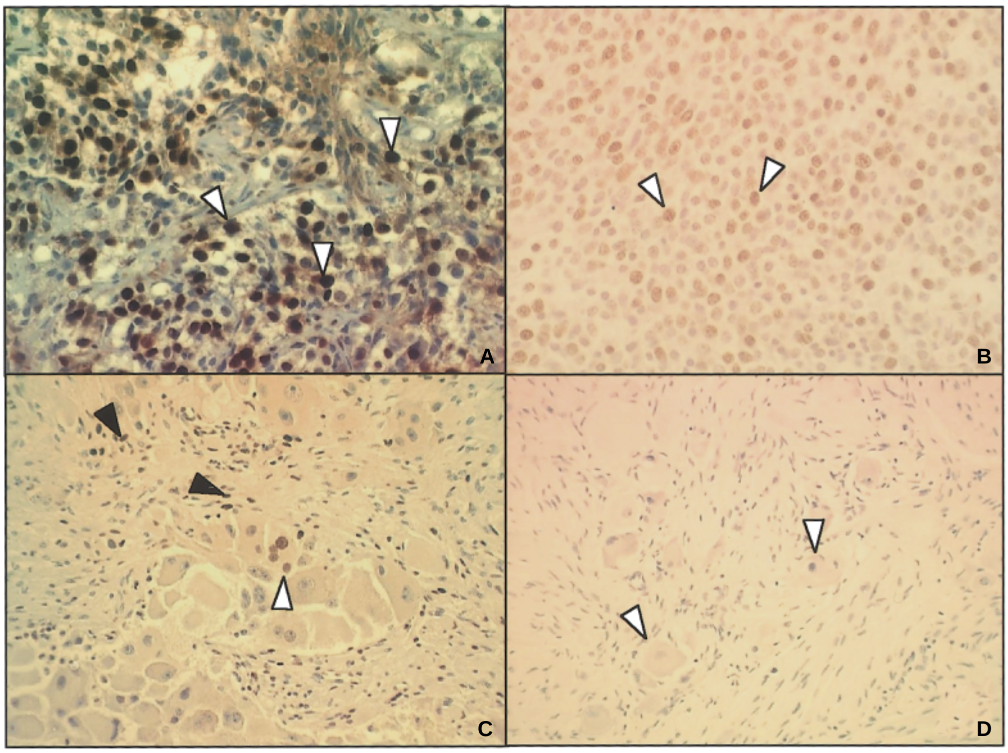

**Figure 3** Immunohistochemical staining for HMGI(Y) in neuroblastic tumours. Nuclear expression of HMGI(Y) in neuroblastic tumours declines as the cells undergo neuronal differentiation. The sections were counterstained with Meyer's haematoxylin. (**A**, **B**) In undifferentiated neuroblastomas, strong nuclear immunoreactivity is found in the majority of the neuroblastic cells (white arrowheads). (**C**) In ganglioneuroblastoma, differentiating neuronal cells (white arrowheads) and some Schwannian cells (black arrowheads) are positive for HMGI-Y while differentiated ganglion cells are negative. (**D**) Significant immunoreactivity is not detected in terminally differentiated ganglionic cells (white arrowheads) of ganglioneuroma. (Magnification: ×200.)

*© 2000 Cancer Research Campaign*

**Table 1** Expression of *HMGI(Y)* in neuroblastic tumours (*n* = 11)

| Case no. | Sex/Age | Stage | Histology | HMGI(Y) | MYCN |
|---|---|---|---|---|---|
| 1 | M/3 mo. | 2 | NB | ++ | non-amplified |
| 2 | M/17 mo. | 4 | NB | +++ | amplified |
| 3 | F/9 mo. | 4 | NB | +++ | non-amplified |
| 4 | M/10 mo. | 3 | NB | ++ | non-amplified |
| 5 | F/19 mo. | 4 | NB | ++ | amplified |
| 6 | F/12 mo. | 2 | GNB | ++ | non-amplified |
| 7 | F/37 mo. | 3 | GNB | + | non-amplified |
| 8 | M/20 mo. | 4 | GNB | + | non-amplified |
| 9 | F/19 mo. | 4 | GN | + | non-amplified |
| 10 | F/60 mo. | 4 | GN | + | non-amplified |
| 11 | F/13 mo. | 4 | GN | + | non-amplified |

mo., months; NB, neuroblastoma; GNB, ganglioneuroblastoma; GN, ganglioneuroma. +: >10–30%, ++: 30–60%, +++: ≥60% (immunoreactivity in undifferentiated or neuronal cells).

In agreement with previous results, the typical nuclear expression of *HMGI(Y)* was found in all the 11 cases analysed (Table 1 and Figure 3). However, we observed marked differences in the immunoreactivity within the different cell populations of the same tumour, and between tumours with different histological appearance. In fact, while we generally observed a strong immunoreactivity in a majority of undifferentiated neuroblastoma cells, we detected a much lower number of immunoreactive cells in more differentiated tumours (Table 1 and Figure 3). The undifferentiated cells in neuroblastomas showed strong and diffuse nuclear staining (Figure 3A, B). In ganglioneuroblastomas, neuronally differentiating cells were positive for HMGI(Y), but immunoreactivity was weaker in cells having more differentiated phenotype (Figure 3C). Occasional Schwann cells were also positive for HMGI(Y) (Figure 3C). The terminally differentiated neuronal cells mostly present in ganglioneuromas were generally negative and only scant neuronal cells showed very weak or hardly discernible immunoreactivity (Figure 3D).

### DISCUSSION

In a previous study we described the expression of HMGI family members in NB cell lines and tumours and suggested their involvement in determining retinoic acid resistance in NB cell lines (Giannini et al, 1999). The work presented here largely extends and completes our previous report, by adding information on the expression of the different HMGI isoforms at both the mRNA and protein level and by including the immunohistochemical characterization of HMGI(Y) expression in neuroblastic tumours, thus providing interesting insights for both NB tumour biology and diagnosis.

NB shows a very high incidence of spontaneous regression and differentiation in infants (Bolande, 1985). Probably reflecting this feature, many cultured NB cell lines can be induced to undergo growth arrest and differentiate by a variety of agents, including RA (Albemayor and Sidell, 1989; Thiele, 1991b). This process is accompanied by the regulation of the expression of several genes at the transcriptional and post-transcriptional level. Repression of c-myc or mycN (in *MYCN* amplified cell lines) (Thiele et al, 1985), increase in Trk receptors (Brodeur et al, 1997) and inhibition of cell cycle progression mostly due to an increase in the cyclin dependent kinase inhibitor p27 (Matsuo and Thiele, 1998), are associated and at least in part responsible for the biological responses of NB cells to RA. Inability to morphologically differentiate into more neuronal phenotypes and attain a complete arrest in the G0 phase of the cell cycle in response to RA has also been described for several NB cell lines. These cells are referred to as *RA-resistant*, even though they are still capable of transducing RA-dependent signals. We have recently shown that RA represses HMGI(Y) mRNA expression in RA-sensitive cells, while it increases HMGI(Y) mRNA in RA-resistant NB cells (Giannini et al, 1999). Here we have confirmed that this increase occurs for both HMGI and HMGY isoforms at the RNA and protein level. We presented also evidence that HMGI-C protein is heavily expressed in RA-resistant cells. Taken together these observations suggest that high levels of HMGI family members may be in part responsible for the resistance to RA-dependent growth inhibition in cells like SK-N-AS or SK-N-SH. Alternatively, since these cell lines do not undergo RA-dependent neuronal differentiation, but still respond to RA with respect to additional biological parameters (i.e., genomic responses), the increase in HMGI and -Y proteins might occur as a part of a growth/differentiation programme alternative to the neuronal differentiation pathway. In any case it is interesting to note that, although HMGI expression is higher than HMGY in all cell lines, RA does not significantly modify the ratio between the two isoforms. This is compatible with the hypothesis that most of the RA effect occurs at the level of transcription of the common unspliced precursor transcript (G. Giannini, unpublished data). Therefore RA appears to act differently than the phorbol esther TPA that preferentially increases HMGY expression in transformation sensitive JB6 cells (Cmarik et al, 1998). Whether HMGI and HMGY perform identical or different biological activities in NB cells or elsewhere, still remains elusive.

In analysing *HMGI(Y)* expression in RA-sensitive cell lines, we have observed that both HMGI and HMGY mRNA are reduced by RA, and that the ratio between the two isoforms is not significantly modified. In contrast, we could not detect a strong decrease at the protein level. The failure to identify a consistent reduction of HMGI and/or HMGY proteins in RA-sensitive cells could be partially explained by the prolonged stability of these molecules (Holth et al, 1997). It is alo possible that HMGI(Y) mRNA reduction by RA is a consequence more than a cause of the growth arrest and neuronal differentiation induced by RA, and that a sudden reduction in HMGI and -Y proteins is not required for these processes to occur. Consistent with this hypothesis, exogenous expression of an HMGI-GFP construct in RA-sensitive SY5Y cells did not affect RA sensitivity (Giannini et al, 1999). In any case, although the biological significance of reduced *HMGI(Y)* expression in more differentiated neuroblastic cells remained

© 2000 Cancer Research Campaign 

elusive thus far, the association between neuroblastic differentiation and low levels of *HMGI(Y)* expression is also confirmed by our immunohistochemical analysis on neuroblastic tumours. In fact, we also show that in vivo, higher *HMGI(Y)* expression is observed in less differentiated phenotypes, while neuronally differentiated cells only have scant expression of *HMGI(Y)*, if any.

High levels of *HMGI(Y)* expression have been associated with advanced grade tumours both in vitro and in vivo. The extensive analysis of its expression in thyroid, colorectal and uterine cervix cancers has indicated its possible role as a diagnostic and possibly as a prognostic factor (Chiappetta et al, 1995, 1998; Fedele et al, 1996; Tamimi et al, 1996; Bandiera et al, 1998; Abe et al, 1999). Stratification of neuroblastic tumour (NT) patients is currently based on the examination of a large panel of clinical, histopathological and molecular parameters, including patient age and stage, levels of serum factors (ferritin, neuron specific enolase, etc.), degree of neuroblastic differentiation and Schwannian/stroma components, presence of *MYCN* amplification, ploidy, chromosome 1p deletions, expression of specific genes like the NGF receptor TrkA, and protein involved in multidrug resistance (MDR1 and MRP) (reviewed in Castleberry, 1997; Katzenstein et al, 1998; Shimada et al, 1999). Although many of these markers have been shown to be predictive of the patient outcome, many of them are not independent. Among the molecular markers, detection of *MYCN* amplification appears to be the most reliable negative prognostic indicator (Seeger et al, 1985; Katzenstein et al, 1998). However, although most patients with favourable histology do not show *MYCN* amplification, a consistent number of unfavourable histology cases also show no *MYCN* amplification, suggesting that additional parameters are still required for a better stratification of NT patients (Castleberry, 1997). We have shown that, similar to other cancers, *HMGI(Y)* is expressed in all NT studied by RT-PCR or immunohistochemistry, with higher expression in less differentiated and more aggressive NB tumours compared to GNB and GN. The higher levels of HMGI(Y) expression seem to be independent from *MYCN* amplification. Therefore, although our observations need to be extended to larger series of patients, they strongly suggest that the expression of *HMGI(Y)* might represent a potential parameter to be evaluated in the stratification of NT patients.

Despite its proven effect on several NB cell lines and its valuable employment in the treatment of a number of malignancies, the efficacy of retinoic acid treatment in NB patients is still controversial (Finklestein et al, 1992; Adamson et al, 1997). As reported in a very recent study, treatment of high-risk neuroblastoma cases with 13-*cis*-RA following either bone marrow transplantation or intense chemotherapy produced a significant amelioration of the 3-year event-free survival (Matthay et al, 1999). However not all patients appeared equally sensitive to RA treatment. Only patients with minimal residual disease benefited from such treatment (Matthay et al, 1999). Our results point out that quantitative and qualitative differences exist in the pattern of expression of the HMGI proteins among different NB cell lines and neuroblastic tumours. Furthermore, high expression of HMGI-C and induction of HMGI(Y) by RA are associated with and partially responsible for NB cell resistance to the antiproliferative effect of RA in vitro. If the association between the increased expression of the HMGI family members and the reduced sensitivity to RA should be confirmed also in vivo, this parameter could help in predicting which cases might benefit from a RA-based therapeutical approach.

### ACKNOWLEDGEMENTS

This work was partially supported by grants from the Associazione per la lotta Neuroblastoma (ANB), the Associazione Italiana per la Ricerca sul Cancro (AIRC), the National Research Council (CNR), Biotechnology Project, the Ministry of University, Research and Technology (MURST, Grant N. 9806279300 and 980626798800), Pasteur Institute Cenci-Bolognetti Foundation and Università degli studi di Trieste.

### REFERENCES

Abe N, Watanabe T, Sugiyama M, Uchimura H, Chiappetta G, Fusco A and Atomi Y (1999) Determination of high mobility group I(Y) expression level in colorectal neoplasias: a potential diagnostic marker. *Cancer Res* **59**: 1169–1174

Adamson PC, Reaman G, Finklestein JZ, Feusner J, Berg SL, Blaney SM, O'Brien M, Murphy RF and Balis FM (1997) Phase I trial and pharmacokinetic study of all-trans-retinoic acid administered on an intermittent schedule in combination with interferon-alpha2a in pediatric patients with refractory cancer. *J Clin Oncol* **15**: 3330–3337

Albemayor E and Sidell N (1989) Human neuroblastoma cell lines as model for the in vitro study of neoplastic and neuronal cell differentiation. *Environ Health Perspect* **80**: 3–15

Arlotta P, Tai AK, Manfioletti G, Clifford C, Jay G and Ono SJ (2000) Transgenic mice expressing a truncated form of the high mobility group I-C protein develop adiposity and an abnormally high prevalence of lipomas. *J Biol Chem* **275**: 14394–14400

Bandiera A, Bonifacio D, Manfioletti G, Mantovani F, Rustighi A, Zanconati F, Fusco A, Di Bonito L and Giancotti V (1998) Expression of HMGI(Y) proteins in squamous intraepithelial and invasive lesions of the uterine cervix. *Cancer Res* **58**: 426–431

Battista S, Fidanza V, Fedele M, Klein-Szanto AJ, Outwater E, Brunner H, Santoro M, Croce CM and Fusco A (1999) The expression of a truncated HMGI-C gene induces gigantism associated with lipomatosis. *Cancer Res* **59**: 4793–4797

Berlingieri MT, Manfioletti G, Santoro M, Bandiera A, Visconti R, Giancotti V and Fusco A (1995) Inhibition of HMGI-C protein synthesis suppresses retrovirally induced neoplastic transformation of rat thyroid cells. *Mol Cell Biol* **15**: 1545–1553

Berner JM, Meza-Zepeda LA, Kools PF, Forus A, Schoenmakers EF, Van de Ven WJ, Fodstad O and Myklebost O (1997) HMGIC, the gene for an architectural transcription factor, is amplified and rearranged in a subset of human sarcomas. *Oncogene* **14**: 2935–2941

Bolande RP (1985) Spontaneous regression and cytodifferentiation of cancer in early life: The oncogenic grace period. *Suev Synth Path Res* **4**: 296–311

Brodeur GM, Nakagawara A, Yamashiro DJ, Ikegaki N, Liu XG, Azar CG, Lee CP and Evans AE (1997) Expression of TrkA, TrkB and TrkC in human neuroblastomas. *J Neurooncol* **31**: 49–55

Bustin M and Reeves R (1996) High-mobility-group chromosomal proteins: architectural components that facilitate chromatin function. *Prog Nucleic Acid Res Mol Biol* **54**: 35–100

Castleberry RP (1997) Neuroblastoma. *Eur J Cancer* **33**: 1430–1437; discussion 1437–1438

Chiappetta G, Bandiera A, Berlingieri MT, Visconti R, Manfioletti G, Battista, S, Martinez-Tello FJ, Santoro M, Giancotti V and Fusco A (1995) The expression of the high mobility group HMGI (Y) proteins correlates with the malignant phenotype of human thyroid neoplasias. *Oncogene* **10**: 1307–1314

Chiappetta G, Avantaggiato V, Visconti R, Fedele M, Battista S, Trapasso F, Merciai BM, Fidanza V, Giancotti V, Santoro M, Simeone A and Fusco A (1996) High level expression of the HMGI (Y) gene during embryonic development. *Oncogene* **13**: 2439–2446

Chiappetta G, Tallini G, De Biasio MC, Manfioletti G, Martinez-Tello FJ, Pentimalli F, de Nigris F, Mastro A, Botti G, Fedele M, Berger N, Santoro M, Giancotti V and Fusco A (1998) Detection of high mobility group I HMGI(Y) protein in the diagnosis of thyroid tumors: HMGI(Y) expression represents a potential diagnostic indicator of carcinoma. *Cancer Res* **58**: 4193–4198

Cmarik JL, Li Y, Ogram SA, Min H, Reeves R and Colburn NH (1998) Tumor promoter induces high mobility group HMG-Y protein expression in transformation-sensitive but not-resistant cells. *Oncogene* **16**: 3387–3396

 *© 2000 Cancer Research Campaign*

Fedele M, Bandiera A, Chiappetta G, Battista S, Viglietto G, Manfioletti G, Casamassimi A, Santoro M, Giancotti V and Fusco A (1996) Human colorectal carcinomas express high levels of high mobility group HMGI(Y) proteins. *Cancer Res* **56**: 1896–1901

Finklestein JZ, Krailo MD, Lenarsky C, Ladisch S, Blair GK, Reynolds CP, Sitarz AL and Hammond GD (1992) 13-cis-retinoic acid (NSC 122758) in the treatment of children with metastatic neuroblastoma unresponsive to conventional chemotherapy: report from the Childrens Cancer Study Group. *Med Pediatr Oncol* **20**: 307–311

Friedmann M, Holth LT, Zoghbi HY and Reeves R (1993) Organization, inducible-expression and chromosome localization of the human HMG-I(Y) nonhistone protein gene. *Nucleic Acids Res* **21**: 4259–4267

Giancotti V, Pani B, D'Andrea P, Berlingieri MT, Di Fiore PP, Fusco A, Vecchio G, Philp R, Crane-Robinson C, Nicolas RH et al. (1987) Elevated levels of a specific class of nuclear phosphoproteins in cells transformed with v-ras and v-mos oncogenes and by cotransfection with c-myc and polyoma middle T genes. *Embo J* **6**: 1981–1987

Giancotti V, Bandiera A, Ciani L, Santoro D, Crane-Robinson C, Goodwin GH, Boiocchi M, Dolcetti R and Casetta B (1993) High-mobility-group (HMG) proteins and histone H1 subtypes expression in normal and tumor tissues of mouse. *Eur J Biochem* **213**: 825–832

Giannini G, Dawson MI, Zhang X and Thiele CJ (1997) Activation of three distinct RXR/RAR heterodimers induces growth arrest and differentiation of neuroblastoma cells. *J Biol Chem* **272**: 26693–26701

Giannini G, Di Marcotullio L, Ristori E, Zani M, Crescenzi M, Scarpa S, Piaggio G, Vacca A, Peverali FA, Diana F, Screpanti I, Frati L and Gulino A (1999) HMGI(Y) and HMGI-C genes are expressed in neuroblastoma cell lines and tumors and affect retinoic acid responsiveness. *Cancer Res* **59**: 2484–2492

Hirning-Folz U, Wilda M, Rippe V, Bullerdiek J and Hameister H (1998) The expression pattern of the Hmgic gene during development. *Genes Chromosomes Cancer* **23**: 350–357

Holth LT, Thorlacius AE and Reeves R (1997) Effects of epidermal growth factor and estrogen on the regulation of the HMG-I/Y gene in human mammary epithelial cell lines. *DNA Cell Biol* **16**: 1299–1309

Ishwad CS, Shriver MD, Lassige DM and Ferrell RE (1997) The high mobility group I-C gene (HMGI-C): polymorphism and genetic localization. *Hum Genet* **99**: 103–105

Johnson KR, Lehn DA and Reeves R (1989) Alternative processing of mRNAs encoding mammalian chromosomal high-mobility-group proteins HMG-I and HMG-Y. *Mol Cell Biol* **9**: 2114–2123

Katzenstein HM, Bowman LC, Brodeur GM, Thorner PS, Joshi VV, Smith EI, Look AT, Rowe ST, Nash MB, Holbrook T, Alvarado C, Rao PV, Castleberry RP and Cohn SL (1998) Prognostic significance of age, MYCN oncogene amplification, tumor cell ploidy, and histology in 110 infants with stage D(S) neuroblastoma: the pediatric oncology group experience – a pediatric oncology group study. *J Clin Oncol* **16**: 2007–2017

Kazmierczak B, Rosigkeit J, Wanschura S, Meyer-Bolte K, Van de Ven WJ, Kayser K, Krieghoff B, Kastendiek H, Bartnitzke S and Bullerdiek J (1996) HMGI-C rearrangements as the molecular basis for the majority of pulmonary chondroid hamartomas: a survey of 30 tumors. *Oncogene* **12**: 515–521

Kottickal LV, Sarada B, Ashar H, Chada K and Nagarajan L (1998) Preferential expression of HMGI-C isoforms lacking the acidic carboxy terminal in human leukemia. *Biochem Biophys Res Commun* **242**: 452–456

Manfioletti G, Giancotti V, Bandiera A, Buratti E, Sautiere P, Cary P, Crane-Robinson C, Coles B and Goodwin GH (1991) cDNA cloning of the HMGI-C phosphoprotein, a nuclear protein associated with neoplastic and undifferentiated phenotypes. *Nucleic Acids Res* **19**: 6793–6797

Matsuo T and Thiele CJ (1998) p27Kip1: a key mediator of retinoic acid induced growth arrest in the SMS-KCNR human neuroblastoma cell line. *Oncogene* **16**: 3337–3343

Matthay KK, Villablanca JG, Seeger RC, Stram DO, Harris RE, Ramsay NK, Swift P, Shimada H, Black CT, Brodeur GM, Gerbing RB and Reynolds CP (1999) Treatment of high-risk neuroblastoma with intensive chemotherapy, radiotherapy, autologous bone marrow transplantation, and 13-cis-retinoic acid. Children's Cancer Group. *N Engl J Med* **341**: 1165–1173

Pedeutour F, Quade BJ, Sornberger K, Tallini G, Ligon AH, Weremowicz S and Morton CC (2000) Dysregulation of HMGIC in a uterine lipoleiomyoma with a complex rearrangement including chromosomes 7, 12, and 14. *Genes Chromosomes Cancer* **27**: 209–215

Rommel B, Rogalla P, Jox A, Kalle CV, Kazmierczak B, Wolf J and Bullerdiek J (1997) HMGI-C, a member of the high mobility group family of proteins, is expressed in hematopoietic stem cells and in leukemic cells. *Leuk Lymphoma* **26**: 603–607

Schoenmakers EF, Wanschura S, Mols R, Bullerdiek J, Van den Berghe H and Van de Ven WJ (1995) Recurrent rearrangements in the high mobility group protein gene, HMGI-C, in benign mesenchymal tumours. *Nat Genet* **10**: 436–444

Seeger RC, Brodeur GM, Sather H, Dalton A, Siegel SE, Wong KY and Hammond D (1985) Association of multiple copies of the N-myc oncogene with rapid progression of neuroblastomas. *N Engl J Med* **313**: 1111–1116

Shimada H, Ambros IM, Dehner LP, Hata J, Joshi VV, Roald B, Stram DO, Gerbing RB, Lukens JN, Matthay KK and Castleberry RP (1999) The International Neuroblastoma Pathology Classification (the Shimada system). *Cancer* **86**: 364–372

Tamimi Y, van der Poel HG, Karthaus HF, Debruyne FM and Schalken JA (1996) A retrospective study of high mobility group protein I(Y) as progression marker for prostate cancer determined by in situ hybridization. *Br J Cancer* **74**: 573–578

Thiele CJ (1991a) Biology of pediatric peripheral neuroectodermal tumors. *Cancer Met Rev* **10**: 3119

Thiele CJ (1991b) Pediatric peripheral neuroectodermal tumors, oncogenes and differentiation. *Cancer Invest* **8**: 629–639

Thiele CJ, Reynolds CP and Israel MA (1985) Decreased expression of N-myc precedes retinoic acid induced morphological differentiation of human neuroblastoma. *Nature* **313**: 404–406

Zhou X, Benson KF, Ashar HR and Chada K (1995) Mutation responsible for the mouse pygmy phenotype in the developmentally regulated factor HMGI-C. *Nature* **376**: 771–774

© 2000 Cancer Research Campaign
